# Dynamic epigenetic mode analysis using spatial temporal clustering

**DOI:** 10.1186/s12859-016-1331-z

**Published:** 2016-12-23

**Authors:** YangLan Gan, Han Tao, Guobing Zou, Cairong Yan, Jihong Guan

**Affiliations:** 1 School of Computer Science and Technology, Donghua University, Shanghai, China; 2 Department of Computer Science and Technology, Tongji University, Shanghai, China; 3 School of Computer Engineering and Science, Shanghai University, Shanghai, China

**Keywords:** Dynamic epigenetic mode, Spatial temporal clustering, Epigenetic modification, Cellular differentiation

## Abstract

**Background:**

Differentiation of human embryonic stem cells requires precise control of gene expression that depends on specific spatial and temporal epigenetic regulation. Recently available temporal epigenomic data derived from cellular differentiation processes provides an unprecedented opportunity for characterizing fundamental properties of epigenomic dynamics and revealing regulatory roles of epigenetic modifications.

**Results:**

This paper presents a spatial temporal clustering approach, named STCluster, which exploits the temporal variation information of epigenomes to characterize dynamic epigenetic mode during cellular differentiation. This approach identifies significant spatial temporal patterns of epigenetic modifications along human embryonic stem cell differentiation and cluster regulatory sequences by their spatial temporal epigenetic patterns.

**Conclusions:**

The results show that this approach is effective in capturing epigenetic modification patterns associated with specific cell types. In addition, STCluster allows straightforward identification of coherent epigenetic modes in multiple cell types, indicating the ability in the establishment of the most conserved epigenetic signatures during cellular differentiation process.

**Electronic supplementary material:**

The online version of this article (doi:10.1186/s12859-016-1331-z) contains supplementary material, which is available to authorized users.

## Background

An epigenome consists of chemical modifications and variations to histones, DNA methylation and other proteins that package the genome [1, 2]. These epigenetic modifications crucially contribute to epigenetic maintenance of chromatin structures and gene expression regulation [3]. There are various interactions among these modifications, which act combinatorially to orchestrate gene expression in different cell types [4]. When heritable from one cell generation to the next, the epigenetic information can bring about lasting changes in gene expression [5, 6].

Embryonic development is a complex process that requires precise gene regulation to govern developmental decisions during cellular differentiation [7, 8]. However, how gene expression is regulated and maintained along developmental transitions remains to be understood. Currently, it is well accepted that transcription factors binding to cis-regulatory sequences coordinately regulate gene expression in response to various environmental cues [9]. On the contrary, the regulatory functions of epigenetic modifications that accompany embryogenesis are largely unexplored. To fully investigate the mechanisms of epigenetic regulation in the cellular differentiation process, extensive research efforts provide genome-wide maps of epigenetic modifications at multiple developmental time points. Human embryonic stem cells were differentiated into a variety of precursor cell types [3, 10], including mesendoderm [11], trophoblast-like cells [12], mesenchymal stem cells [13] and neural progenitor cells [14]. Mouse embryonic stem cells were also differentiated into mesendoderm cells [15]. The availability of these temporal epigenomic data provides a unique opportunity for characterizing fundamental properties of epigenome dynamics and revealing regulatory roles of epigenetic modifications.

To establish combinatorial patterns of epigenetic modification, previous computational methods primarily utilize spatial information of epigenetic marks. For example, Chromasig was designed to study histone modification patterns using correlations of histone signals [16]. CoSBI also used the correlations within 5Kbp genomic segments to exhaustively searched for histone code [17]. ChromHMM applied a HMM model to annotate genomic sequences by co-occurrence of multiple epigenetic marks [18]. RFECS was developed based on random forests [19]. AWNFR explored epigenomic landscapes using the wavelet transforms [20]. Although these methods successfully identify the combinatorial epigenetic mode based on spatial epigenomic information, there is still an urgent need to exploit newly produced temporal information to study the dynamic patterns and functions of epigenetic modifications.

In this study, we developed a spatial temporal clustering approach that exploits the temporal variation information of epigenomes along the differentiation process, aiming to characterize dynamic properties of epigenetic modifications. This approach identifies significant spatial temporal patterns of epigenetic modifications during embryogenesis and cluster regulatory sequences by their spatial temporal epigenetic patterns. The results might shed a light on how epigenetic modifications evolve temporally and how the spatial temporal patterns of epigenetic modifications regulate gene expression during the process of cellular differentiation.

## Methods

### Datasets

In mammals, studying the epigenetic mechanisms of early embryonic development often requires access to embryonic cell types. In recent studies, to analyze early human developmental decisions, human embryonic stem cells (hESCs) were differentiated into trophoblast-like cells, mesendoderm, mesenchymal stem cells, and neural progenitor cells [3, 10]. The first three states represent developmental events that mirror critical developmental decisions in the embryo. Mesenchymal stem cells are fibroblastoid cells that are capable of expansion and multi-lineage differentiation to bone, cartilage, adipose, muscle, and connective tissues. In these cell types, genome-wide maps of main epigenetic marks have been generated using ChIP-seq [21]. In detail, the investigated epigenetic modifications were profiled, including H3K4me1/2/3, H3K36me3, H3K9me3, H3K27me3, H3K79me1, H2AK5ac, H2bK120ac, H2BK5ac, H3K18ac, H3K23ac, H3K27ac, H3K4ac, H3K9ac and H4K8ac. RNA expression profiles of these five cell types were also generated using Affymetrix GeneChip-arrays. Here, we downloaded these datasets from the website of NIH Roadmap Epigenome Project (http://www.epigenomebrowser.org/) [22].

### Methods

### General scheme of the STCluster algorithm

**Fig. 1:**
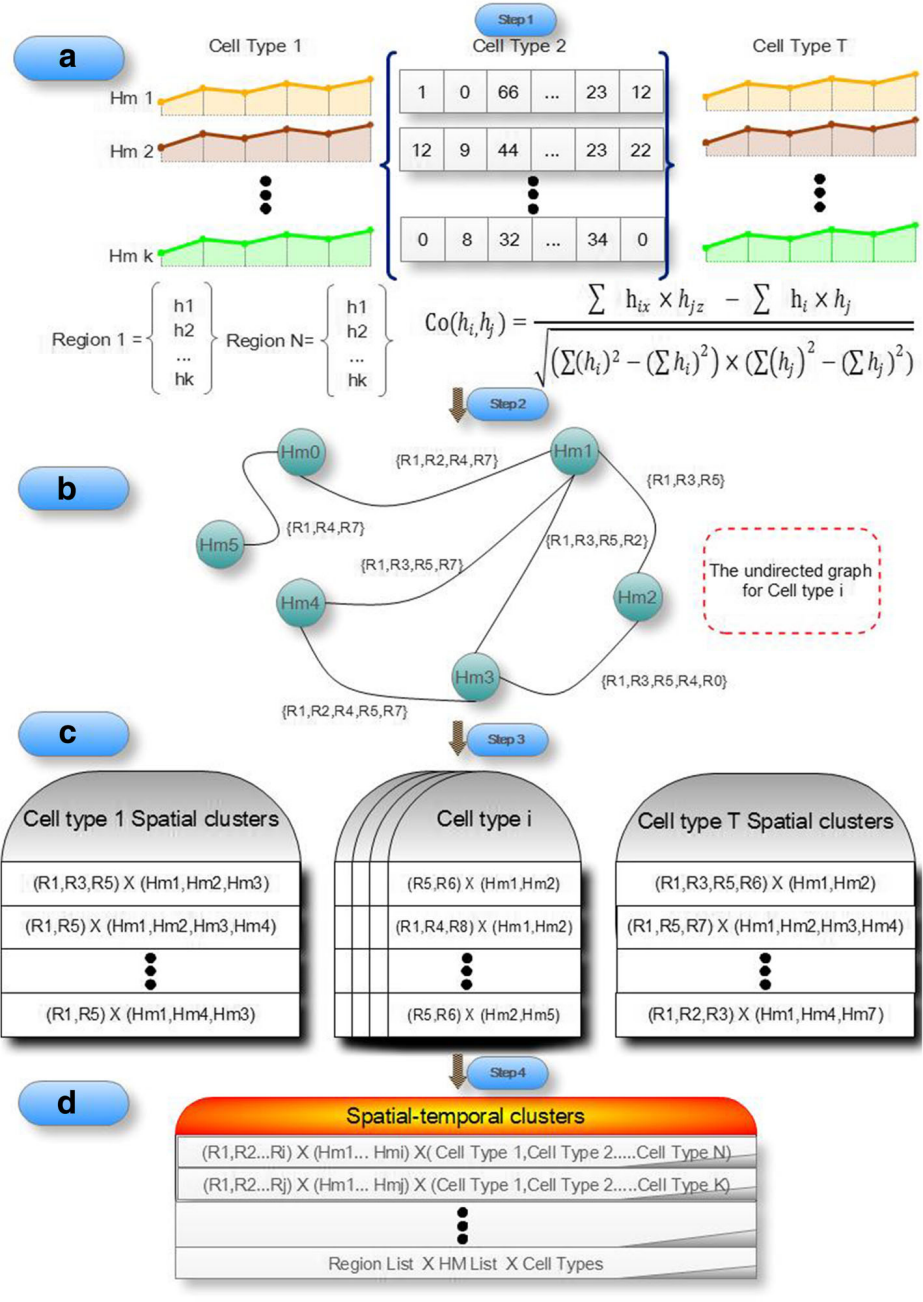
The overview of STCluster algorithm. **a** Step 1, ChIP-seq data transformation. **b** Step 2, construct the co-occurrence graph for each cell type. **c** Step 3, mine the co-occurrence graphs to identify spatial clusters with coherent epigenomic modification patterns. **d** Step 4, identify spatial-temporal clusters during the differentiation process

The STCluster algorithm analyzes genome-wide maps of epigenetic modifications to characterize dynamic epigenetic signatures during embryonic stem cell differentiation. There are four major steps in the STCluster algorithm: (i) ChIP-seq data transformation, (ii) construct the co-occurrence graph for each cell type, (iii) mine the co-occurrence graphs to identify spatial clusters of genomic segments with coherent epigenetic patterns, and (iv) mine the resulted spatial clusters of each cell type to identify spatial temporal clusters and discover conserved epigenetic signatures during the differentiation process. Figure 1 illustrates the scheme of the STCluster algorithm. In the following, we elaborate the process of epigenetic mode analysis step by step.

#### Step 1. Data transformation

The whole human genome were divided into non-overlapping 200bp bins. For each epigenetic modification map, we first computed the summary tag count of every bin. Then, in each cell type, raw sequence read counts of each epigenetic modification were normalized by the total number of reads followed by arcsine transformation [23], to remove noises resulting from spurious tag counts in the ChIP-seq experiments. Further, we divided the whole genome into 5Kbp genomic regions. In this way, for each cell type, the profiles of these epigenetic modifications are represented as a matrix *R*_*i*_, where i is the index of the genomic regions ranging from 1 to N (assuming there are totally N genomic regions under consideration), as shown in Fig. 1 a. In each region, the number of columns is denoted as B and the number of epigenetic modifications is denoted as K. The column vectors correspond to combinatorial epigenetic modification tag counts within individual genomic bins and the row vectors correspond to the contiguous genomic landscape of individual epigenetic modifications.

#### Step 2. Construct co-occurrence graph for each cell type

In this step, we computed the correlation coefficients of epigenetic modification pairs in each region, and then we constructed the corresponding co-occurrence graph for each cell type. Given the processed and organized epigenetic modification data of each cell type, correlation coefficients of any two histone modifications at every region were calculated to obtain a coefficient matrix. If the coefficients are higher than a given threshold, the two epigenetic modifications are regarded as coherent in this region. Subsequently, this region was added to the corresponding region set. Based on the coefficient matrix, we further constructed the co-occurrence graph, which is modeled as an undirected graph G = (V, E), where V is the set of all histone modifications. For any two epigenetic modification types *h*_*i*_ and *h*_*j*_ (i ≠j), if they are correlated at any region, there exists an edge *e*∈*E* between vertices *h*_*i*_ and *h*_*j*_. In addition, each edge in the co-occurrence graph is associated with the region set. Figure 1 b shows an example of co-occurrence graph. Here, we set the correlation coefficient threshold as 0.9 to achieve a high quality of spatial clusters.

#### Step 3. Mine spatial clusters from co-occurrence graph

The co-occurrence graph represents in a compact way all the correlated epigenetic modifications in different regions. It can be used to mine potential spatial clusters corresponding to each developmental stage, and filter out most of the unrelated data. The STCluster algorithm applies a depth first search (DFS) strategy on the co-occurrence graph to mine all the spatial clusters. A typical spatial cluster represents a group of genomic regions that share spatial epigenetic patterns. To gain the significant epigenetic states, we set the minimum number of histone modifications as 5 and the minimum percent of regions as 0.1%. For each cell type, we identified a set of spatial clusters, as shown in Fig. 1 c.

#### Step 4. Identify spatial-temporal clusters from spatial clusters

On obtaining the maximal spatial cluster set for all cell types, we utilized them to mine the maximal spatial-temporal clusters. This is accomplished by enumerating the subsets of the time points (Fig. 1 d), using a process similar to the spatial cluster clique mining. The regions in each spatial temporal clusters exhibit similar changes of epigenetic modifications during the cellular differentiation process. Spatial-temporal clusters indicate specific conserved chromatin signatures that are shared by multiple time points along the embryonic stem cell differentiation.

## Results and Discussion

### Identifying combinatorial epigenetic states during differentiation

To investigate combinatorial epigenetic states during the differentiation of embryonic stem cells, we applied STCluster to the genome-wide epigenetic modification maps of five cell types, including H1, Mesendoderm, Trophoblast-like cells, Mesenchymal stem cells and Neuronal progenitor cells. STCluster first grouped genomic regions based on spatial patterns of epigenetic modifications to identify spatial clusters. For each cell type, we set the minimum number of histone modifications as 5 and the minimum percent of regions as 0.1%, which allow us to capture patterns that involve at least five epigenetic modifications and re-occur across at least 0.1% of the human genome. With this parameter setting, we respectively identified 3344, 667, 4726, 1422, 1984 spatial clusters in H1, Mesendoderm, Trophoblast-like cells, Mesenchymal stem cells and Neuronal progenitor cells.

**Fig. 2:**
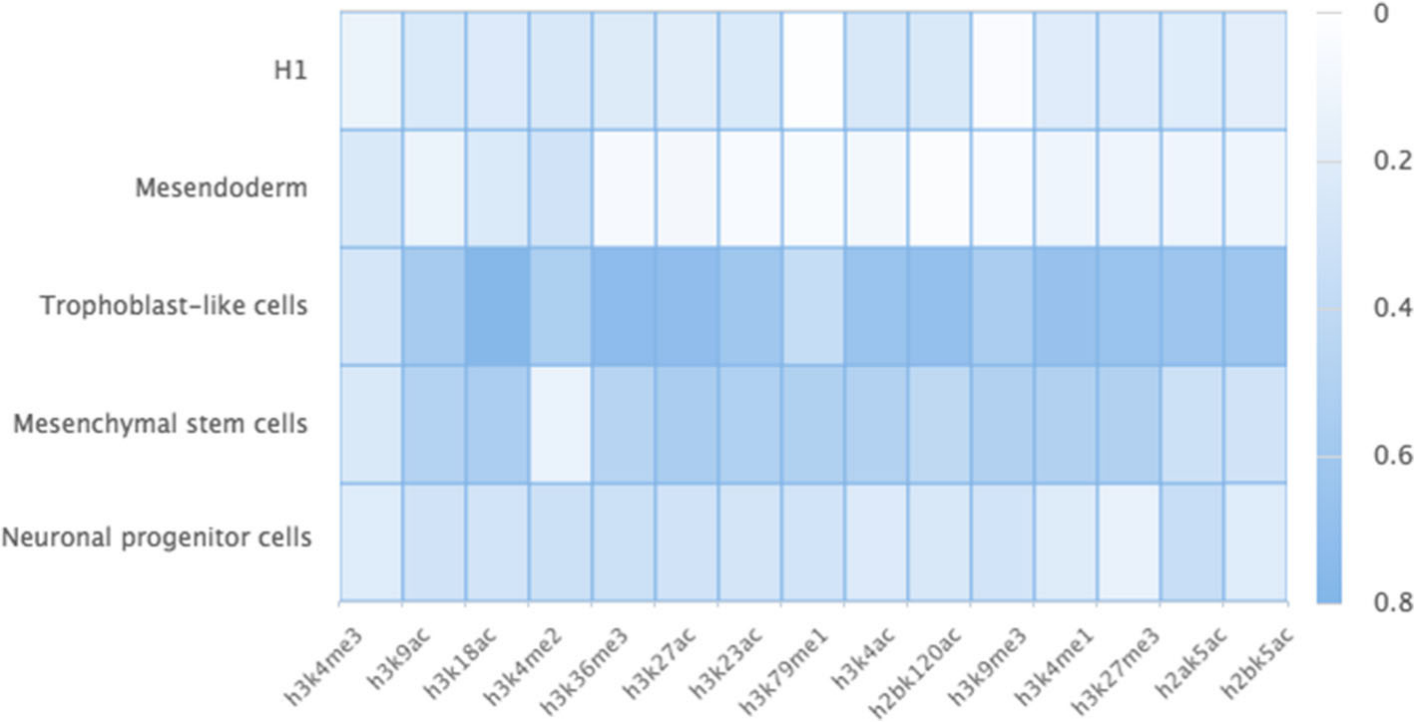
The occurrence frequencies of all investigated epigenetic modifications in the identified spatial clusters

Next, we evaluated the occurrence frequencies of all investigated epigenetic modifications in the identified spatial clusters. Specifically, the occurrence frequency of an epigenetic modification is computed as the ratio of the occurrence in these spatial clusters and the total number of 5Kbp non-overlapping regions in the genome. Their occurrence frequencies are depicted in Fig. 2. We found that epigenetic modification H3K18ac has a high frequency in all cell types, which indicates that these regions share the variation pattern of this epigenetic modification. Epigenetic modifications (H3K4me1/3) seldom occur in the spatial clusters of H1, whereas epigenetic modification (H3K4me2) frequently occur in the spatial clusters of cell types except H1. In the spatial clusters of Neuronal progenitor cells, most epigenetic modifications have median occurrence frequencies.

Expanding this research, we studied co-occurred epigenetic modifications at each developmental stage of the differentiation process. For each cell type, we ranked the identified spatial clusters according to the number of regions co-occupied by epigenetic modification set. We discovered groups of epigenetic modifications that frequently co-occur in each cell type. Table 1 summarizes the top 10 frequently co-occurred epigenetic modifications. Overall, the clustering results show that different cell types exhibit diverse cell type specific patterns of epigenetic modifications. The overlaps of epigenetic modifications among these cell types are small. However, We found that epigenetic marks <H3K4me1, H3K4me2 > and <H3K4me1, H3K4ac > frequently co-occur in the spatial clusters of different cell types. Part of these cell types, such as H1 cell line and Mesendoderm, Mesenchymal stem cells and Neuronal progenitor cells, share more epigenetic patterns than other groups of cell types.

**Table 1:** The top 10 co-occurred epigenetic modifications in different cell types during the differentiation process

	H1	Mesendoderm	Trophoblast-like cells	Mesenchymal stem cells	Neuronal progenitor cells
1	H3K4me2,H3K4me1, H2aK5ac,H3K27ac, H2bK5ac,H3K23ac, H3K9me3,H3K79me1, H3K4ac,H3K9ac, H2bK120ac	H3K4me2,H3K4me1, H3K27ac,H3K23ac, H3K9ac,H3K18ac	H2aK5ac,H2bK5ac, H3K27me3,H3K23ac, H3K9me3,H3K79me1, H3K4ac,H3K9ac, H2bK120ac,H3K18ac	H3K4me2,H3K36me3, H3K27ac,H2bK5ac, H3K23ac,H3K9me3, H3K4ac,H3K9ac, H2bK120ac	H3K4me1,H3K36me3, H2aK5ac,H3K27ac, H2bK5ac,H3K27me3, H3K23ac,H3K79me1, H3K9ac,H2bK120ac
2	H2aK5ac,H2bK5ac, H3K23ac,H3K79me1, H3K4ac,H2bK120ac	H3K4me2,H3K4me1, H3K27ac,H3K23ac, H3K9ac,H2bK120ac	H3K4me1,H2bK5ac, H3K27me3,H3K23ac, H3K9me3,H3K79me1, H3K4ac,H3K9ac, H2bK120ac,H3K18ac	H3K4me2,H3K36me3, H2bK5ac,H3K23ac, H3K4ac,H2bK120ac	H3K4me1,H3K36me3, H3K27ac,H2bK5ac, H3K27me3,H3K9me3, H3K79me1,H3K9ac, H2bK120ac
3	H2bK5ac,H3K27me3, H3K23ac,H3K79me1, H3K4ac,H3K9ac	H2aK5ac,H2bK5ac, H3K79me1,H3K4ac, H2bK120ac,H3K18ac	H3K27ac,H2bK5ac, H3K27me3,H3K23ac, H3K9me3,H3K18ac	H3K36me3,H2aK5ac, H2bK5ac,H3K23ac, H3K9me3,H3K4ac H3K9ac	H3K4me2,H2aK5ac, H3K23ac,H3K9me3, H3K4ac,H2bK120ac
4	H3K4me2,H3K4me1, H2aK5ac,H3K27ac, H2bK5ac,H3K27me3, H3K23ac,H3K79me1, H3K4ac,H3K9ac,H3K18ac	H3K4me1,H3K36me3, H3K27ac,H3K23ac, H3K79me1,H3K4ac, H3K9ac,H2bK120ac, H3K18ac	H3K27ac,H2bK5ac, H3K27me3,H3K23ac, H3K9me3,H2bK120ac	H3K36me3,H2aK5ac, H2bK5ac,H3K23ac, H3K4ac,H3K9ac	H3K4me2,H2aK5ac, H3K23ac,H3K9me3, H3K9ac,H2bK120ac
5	H2bK5ac,H3K27me3, H3K23ac,H3K79me1, H3K4ac,H3K18ac	H3K27ac,H3K27me3, H3K9me3,H3K4ac, H2bK120ac,H3K18ac	H3K27ac,H2bK5ac, H3K27me3,H3K23ac, H3K9me3,H3K9ac	H3K4me2,H3K36me3, H2bK5ac,H3K23ac, H3K9me3,H3K9ac	H3K27ac,H2bK5ac, H3K27me3,H3K9me3, H3K9ac,H2bK120ac
6	H2aK5ac,H2bK5ac, H3K9me3,H3K79me1, H3K4ac,H2bK120ac	H3K4me2,H3K27ac, H3K9me3,H3K4ac, H2bK120ac,H3K18ac	H3K27ac,H2bK5ac, H3K27me3,H3K23ac, H3K9me3,H3K79me1	H3K36me3,H2aK5ac, H2bK5ac,H3K9me3, H3K4ac,H2bK120ac	H3K27ac,H2bK5ac, H3K27me3,H3K9me3, H3K4ac,H2bK120ac
7	H2aK5ac,H2bK5ac, H3K23ac,H3K79me1, H3K4ac,H3K9ac	H3K4me2,H3K4me1, H3K27ac,H3K27me3, H3K9me3,H3K18ac	H3K27ac,H2bK5ac, H3K27me3,H3K23ac, H3K9me3,H3K4ac	H3K4me2,H3K36me3, H2bK5ac,H3K23ac, H3K9me3,H3K4ac	H3K4me2,H3K4me1, H3K23ac,H3K9me3, H3K9ac,H2bK120ac
8	H3K4me2,H2aK5ac, H3K27ac,H2bK5ac, H3K27me3,H3K23ac, H3K9me3,H3K79me1, H3K4ac,H3K9ac,H3K18ac	H3K4me2,H3K4me1, H3K27ac,H3K27me3, H3K4ac,H3K18ac	H3K4me2,H3K27ac, H3K27me3,H3K23ac, H3K9me3,H3K9ac	H3K4me2,H3K36me3, H2bK5ac,H3K23ac, H3K4ac,H3K9ac, H2bK120ac	H3K4me2,H3K4me1, H3K23ac,H3K9me3, H3K4ac,H2bK120ac
9	H3K4me2,H3K4me1, H2aK5ac,H3K27ac, H2bK5ac,H3K23ac, H3K9me3,H3K79me1, H3K4ac,H3K9ac H3K18ac	H3K4me2,H3K4me1, H3K27ac,H3K27me3, H3K4ac,H2bK120ac	H3K4me1,H3K27ac, H3K27me3,H3K23ac, H3K9me3,H3K79me1	H3K4me2,H3K4me1, H3K9me3,H3K4ac, H3K9ac,H2bK120ac	H3K4me2,H3K4me1, H3K36me3,H2aK5ac, H3K27me3,H3K23ac, H3K79me1,H3K9ac, H2bK120ac
10	H2aK5ac,H2bK5ac, H3K9me3,H3K79me1, H3K4ac,H3K9ac	H3K4me2,H3K27ac, H3K27me3,H3K4ac, H2bK120ac,H3K18ac	H3K4me1,H3K27ac, H3K27me3,H3K23ac, H3K9me3,H3K79me1	H3K4me2,H3K36me3, H2bK5ac,H3K23ac, H3K9me3,H2bK120ac	H3K4me2,H3K4me1, H2aK5ac,H3K27ac, H3K9me3,H3K9ac, H2bK120ac

### Identifying conserved epigenetic states during the differentiation of ES cells

**Fig. 3:**
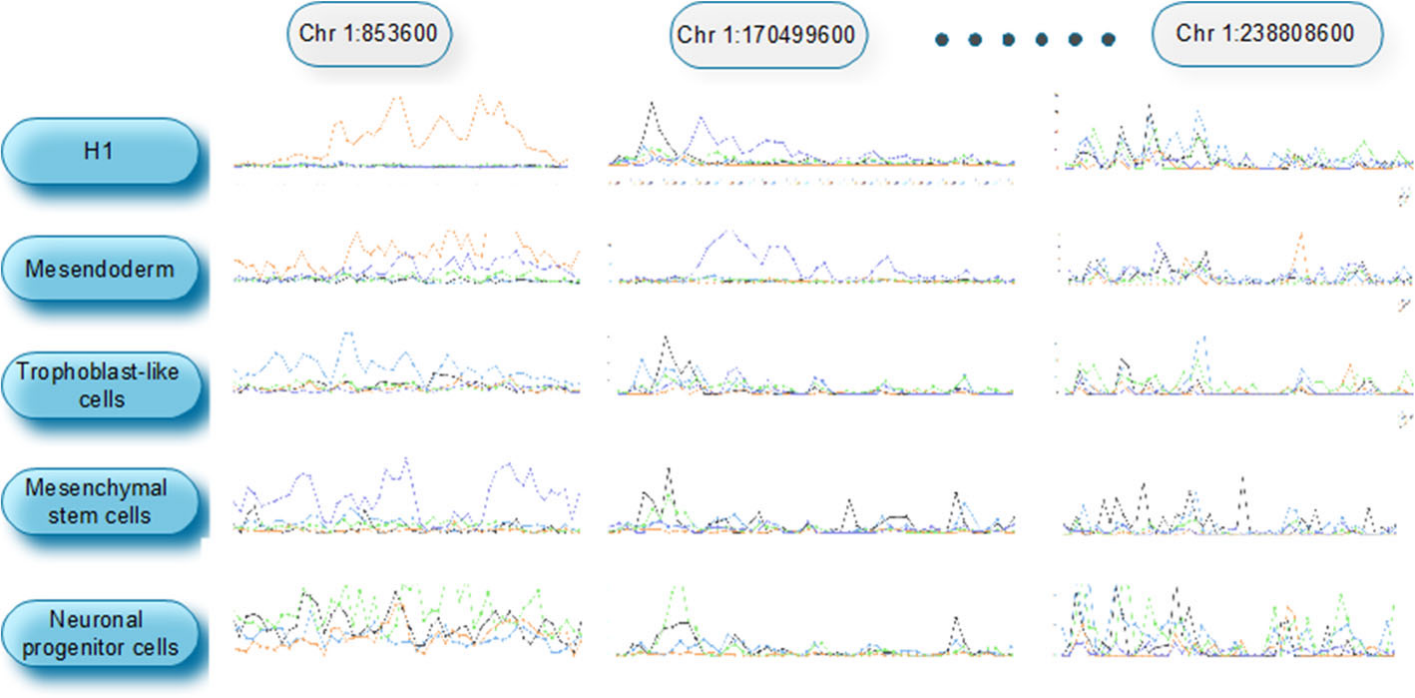
Profiles of co-occurred epigenetic modifications display an overall conserved pattern at five different stages during the differentiation process

There are large differences among the investigated epigenetic modifications regarding their temporal variations. To identify conserved epigenetic states and explore the temporal patterns of these epigenetic modifications, we applied STCluster to further group genomic regions based on the spatial clusters. The identified spatial temporal clusters are represented as triples (<genomic regions >, <epigenetic modifications >, <cell types >). Each cluster lists the genomic regions with the co-occupied epigenetic modifications, which exhibit little variation at different cell types during the differentiation process. Taking a typical spatial temporal cluster as an example, Fig. 3 displays the profiles of co-occurred epigenetic modifications in different regions during the differentiation process. In this cluster, eight epigenetic modifications (H3K4me2, H3K23ac, H3K27ac, H2BK120ac, H3K27me3, H3K79me1, H3K4ac and H3K9ac) co-occur the clustered regions in five different cell types. For each region, these epigenetic marks display conserved modification patterns at all these five stages.

The detailed information of all identified spatial temporal clusters are listed in Additional file 1. The results indicate that there exist conserved epigenetic states during the differentiation process. We observed a high co-occurrence and stable patterns of H3K4me2 with H3K23ac, H3K18ac, H3K27ac and H3K9ac at five different stages along the differentiation process. Our observation is consistent with the previous finding that H3K4me2 is one of the backbone epigenetic modifications along with H3K27ac and H3K9ac [18, 24]. On the contrary, some epigenetic modification patterns are only coherent in certain cell types. For example, the variation pattern of epigenetic modifications <H3K27me3, H3K9me3, H3K79me1, H3K4ac, H3K9ac > is shared in four cell types except Mesendoderm. <H3K4me2, H3K23ac, H3K27ac, H3kK27me3, H3K79me1, H3K18ac > are only shared in H1, Mesendoderm, and Trophoblast-like cells except Mesenchymal stem cells and Neuronal progenitor cells. Notably, the identified spatial temporal clusters reveal more details of the differentiation process.

### Analyzing the regulatory roles of epigenetic modifications during differentiation

**Fig. 4:**
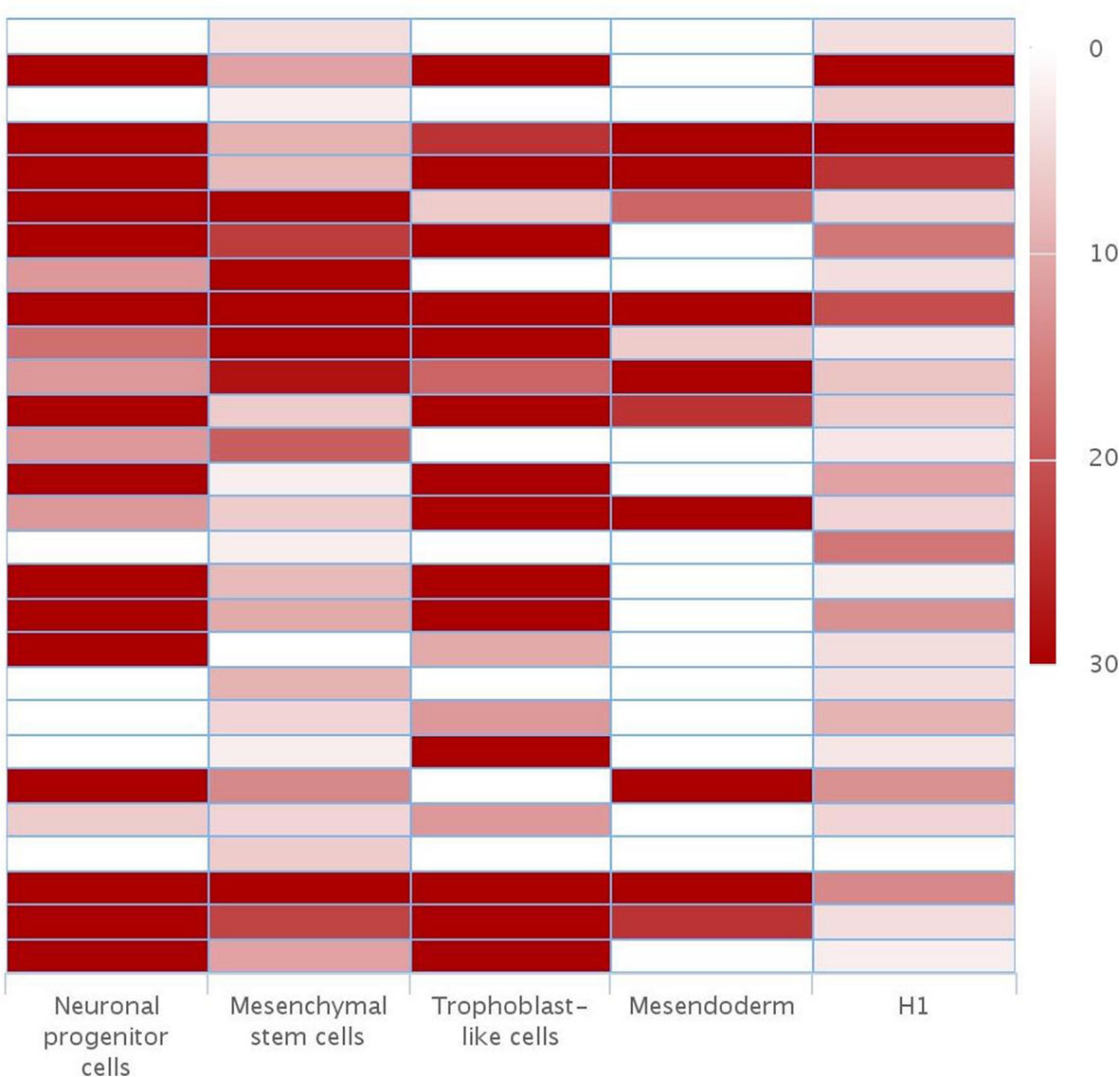
The RNA expression levels of the identified spatial temporal clusters

As epigenetic marks were thought to be predictive of gene expression levels in a context-independent manner [25], we further analyzed the RNA expression levels of the identified spatial temporal clusters to see if this theory holds during embryonic stem cell differentiation. Specifically, we extracted the RNA expression data of the genomic regions included in the spatial temporal clusters, and compared the expression level in the corresponding cell types. Consistent with previous study, some epigenetic marks, such as H3K27ac and H3K36me3, are correlated with RNA expression level of different genomic regions at different developmental stages. However, several epigenetic modifications show cell type specific regulation on mRNA expression. As shown in Fig. 4, the variations of temporal epigenetic modifications are not correlated with gene expression changes in several spatial-temporal clusters, which are primarily located in promoter regions. These results imply that epigenetic patterns mediate gene regulation during cell differentiation in a complex way, rather than in a linear manner.

## Conclusions

Identifying epigenomic dynamics is important to understand mechanisms for gene regulation. Our knowledge about the temporal patterns of epigenetic modifications and the consequence of them are still limited. There is a urgent need to develop new computational approach that exploits the complex epigenomic landscapes and discovers significant signatures out of them. In this study, we developed a spatial temporal clustering algorithm to explore the epigenomic landscapes of five cell types during embryonic stem cell differentiation. Using this approach, we identified spatial temporal patterns of epigenetic modifications in early embryogenesis. Different from previous computational methods, our approach is designed to investigate the dynamic epigenetic landscapes as well as the combinational epigenetic modes. The experimental results demonstrate that the proposed STCluster algorithm could successfully capture dynamic epigenetic modification patterns associated with specific cell types. In addition, STCluster allows straightforward identification of epigenetic conservation at multiple developmental stages during cell differentiation process.

## Additional file

Additional file 1 The detailed information of identified spatial temporal clusters. Details for the spatial temporal clusters, represented as triples (<genomic regions >, <epigenetic modifications >, <cell types >). Each cluster lists the genomic regions with the co-occupied epigenetic modifications, which exhibit little variation at different cell types during the differentiation process. (TXT 3962 kb)
